# Utility of Portable Fundus Camera in Teaching Direct Ophthalmoscopy to Medical Students

**DOI:** 10.1111/tct.70093

**Published:** 2025-04-11

**Authors:** Ching‐Kit Tsui, Yuxian Zou, Zhenzhen Liu, Yiqing Li, Rongxin Chen

**Affiliations:** ^1^ Guangdong Provincial Key Laboratory of Ophthalmology and Visual Science State Key Laboratory of Ophthalmology, Zhongshan Ophthalmic Center, Sun Yat‐sen University Guangzhou China

**Keywords:** direct ophthalmoscope, fundus, medical education, portable fundus camera

## Abstract

**Background:**

Direct ophthalmoscopy (DO) an essential skill for junior doctors. DO allows for quick and effective fundus examinations to detect life‐ and vision‐threatening diseases. However, medical students often find it difficult to master DO due to the challenges associated with fundus visualisation. Innovative and effective approaches are needed at the undergraduate level to improve the acceptance and proficiency of DO.

**Approach:**

In 2023–2024, 79 fourth‐year medical students participated in a study to evaluate the acceptance and effectiveness of a new teaching approach using a portable fundus camera (PFC) during weekly ophthalmology clerkships. This study compared the PFC‐assisted teaching method with the traditional DO teaching method.

**Evaluation:**

**The participants were allocated into two groups, with 40 in Group A and 39 in Group B.** They attended traditional DO and PFC‐assisted modules separately in session one and then crossed over in session two. Questionnaires evaluated perceived ease of use (PEOU), perceived usefulness (PU), confidence and satisfaction at the end of the sessions. All students completed the DO performance using standard patient and fundus photointerpretation assessments and compared with previous non‐intervention group.

**Results:**

The integration of PFC‐assisted teaching into the ophthalmology curriculum effectively addressed traditional barriers to learning DO, fostering confidence and skill acquisition in medical students. The blended approach of combining technology with traditional teaching facilitated a more comprehensive learning experience.

**Implications:**

PFC‐assisted teaching provided an innovative and effective strategy for improving DO training. We would advocate for similar approach may help students to find out the zone of proximal development.

## 1 Background

The ramifications of eye health and vision permeate extensively through all aspects of life [1]. Ophthalmic issues are common in various clinical environments [2]. A strategy to contend with the burden of eye care would strengthen ophthalmic skills for all future frontline medics.

Ophthalmologists are not immediately accessible in most of healthcare settings, especially in China. Direct ophthalmoscopy (DO) is an indispensable competency armamentarium for doctors because it can provide quick and effective fundus examinations to detect life‐ and vision‐threatening diseases. However, some argue that the role of DO is diminishing due to the advent of various digital diagnostic devices. Unfortunately, DO skills also fade during undergraduate medical training, largely due to a lack of clinician proficiency in teaching and practice. Although the art of fundoscopy has been forgotten [3], DO is still recommended as the most readily accessible instrument in most clinical settings [4, 5, 6].

Traditional pedagogical approaches to teaching DO skills involve many challenges [7]. One of the key challenges, leading to a lack of confidence, is the visualisation and identification of fundal structures because of the magnified and narrow field of view. Uncertainty in the interpretation of findings is another challenge. Although medical students could obtain the limited fundal view, they poorly interpreted the fundal features as futile [8]. Furthermore, the extended face‐to‐face nature of novice training contributes to discomfort among students, diminishing their motivation and interest in practicing DO techniques. Digital health has become a trend in the recent decades. Early studies showed that fundus camera and smartphone fundoscopy seem favourable to students compared with DO, with a shorter learning curve and confidence, because students are familiar with the use of these technologies [8, 9, 10]. These digital fundoscopies showed potential educational value for period of apprenticeship.

DO is the gold standard for fundus assessment. From a pedagogical perspective, the current digital camera acts as a potential pedagogical tool for medical students. Therefore, this study aimed to evaluate the change in acceptance of DO by conducting a survey on perceived ease of use (PEOU) and perceived usefulness (PU) of DO [11]. The effectiveness of a portable fundus camera (PFC)‐assisted DO teaching approach compared to traditional direct ophthalmoscopy (TDO) in learning fundus examination skills. We hypothesised that the PFC‐assisted DO teaching method would outperform the TDO teaching method and help students to find out the zone of proximal development.

*Direct ophthalmoscopy (DO) is an indispensable competency armamentarium for doctors because it can provide quick and effective fundus examinations to detect life‐ and vision‐threatening diseases*.

## 2 Approach

Fourth‐year undergraduates, at the Zhongshan School of Medicine, Sun Yat‐sen University, participated in this study in the academic year 2023–2024, totalling 79 individuals enrolled. They had attended regular lectures in ophthalmology without any prior ophthalmic clinical exposure. In this study, Optomed Aurora PFC (Optomed Oy, Oulu, Finland) and 66Vision YZ11 DO (66Vision Tech Co., Ltd, Suzhou, China) were used in the practice.

### 2.1 Group Allocation and Training Design

**FIGURE 1:**
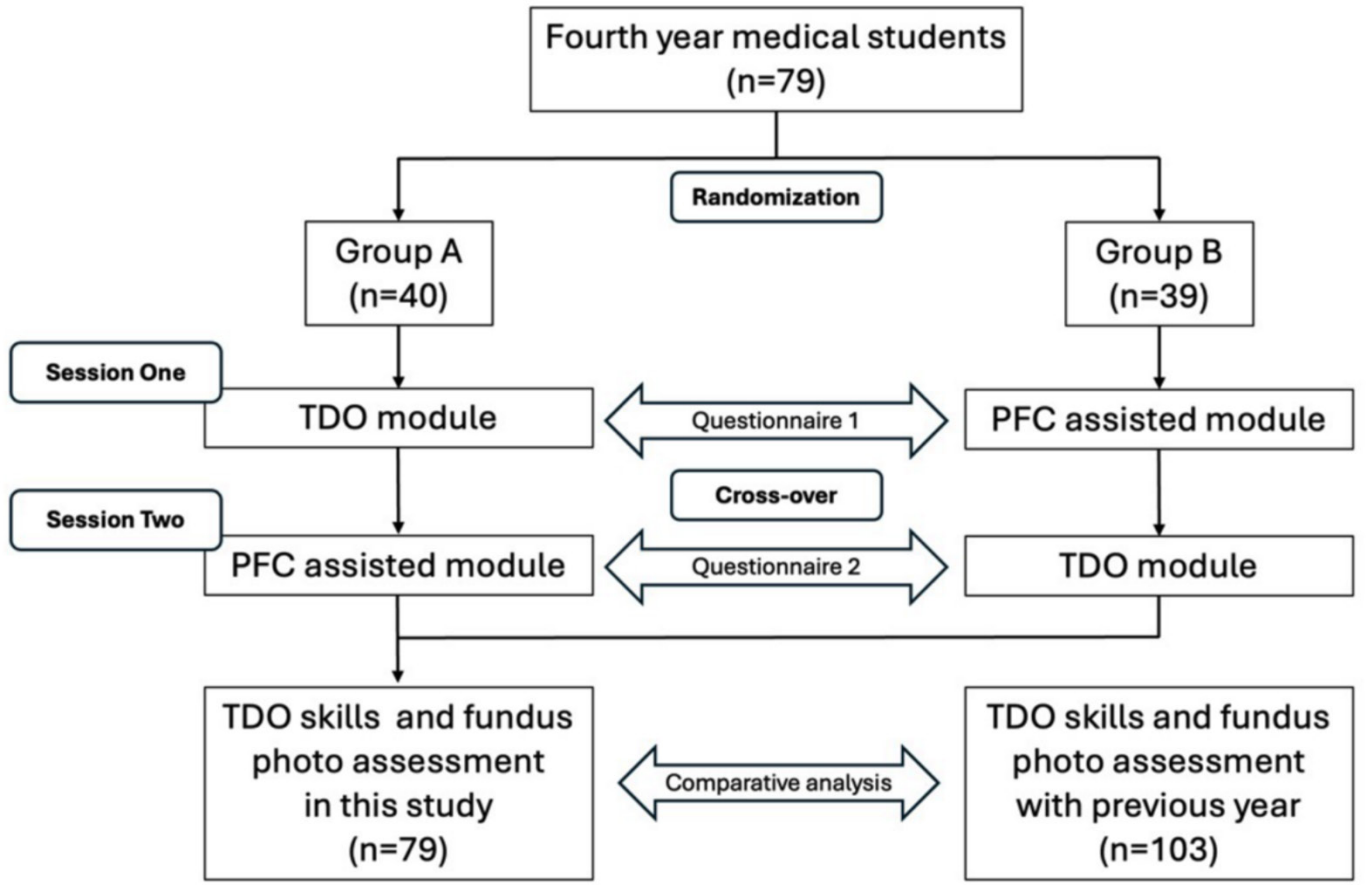
Study flow chart.

The participants were evenly allocated into two groups (40 in Group A and 39 in Group B). The flow chart is shown in Figure 1. In session one, Group A attended the TDO module, while Group B attended the PFC‐assisted teaching module. For fairness, the training was crossed over in session two. Each module consisted of a 10‐min lecture with demonstration and a 50‐min practice under the supervision of two ophthalmologists. The PFC module included an additional 5‐min demonstration by a trained ophthalmologist. Students captured fundus photos of their peers and interpreted the findings, followed by DO practice on both eyes with and without mydriasis.

### 2.2 Assessment of Student Performance

Finally, students completed DO performance and fundus photointerpretation assessments for subjective measures. DO performance was evaluated by fundus specialists based on the ability to visualise the fundus, perform the procedure and interpret findings within 5 min. Photointerpretation was evaluated during the final clerkship examination, focusing on identifying normal and pathological findings.

*Each module consisted of a 10‐min lecture with demonstration and a 50‐min practice under the supervision of two ophthalmologists. The PFC module included an additional 5‐min demonstration by a trained ophthalmologist*.

## 3 Evaluation

At the end of each session, both groups completed a 5‐point Likert scale questionnaire. After crossover, the questionnaire was completed in the same way. The questionnaire assessed DO training (PEOU, PU, self‐satisfaction and confidence) and PFC assistance (satisfaction) (Detailed in Additional file 1). Data from 79 participants were included in the analysis, with 100% (*n* = 40) responses from Group A and 100% (*n* = 39) responses from Group B. The internal reliability of the questionnaire is high (Cronbach's α = 0.953). Gender and age showed no significant differences between the groups (Table 1).

**TABLE 1:** Baseline characteristics of participants in the study.

	Group A (*n* = 40)	Group B (*n* = 39)	Total (*n* = 79)	*p* *
Age (years) (mean ± SD)	21.1 ± 0.34	21.1 ± 0.47	21.1 ± 0.41	0.502
Gender (M:F)	27:12	27:13	54:25	0.200

* Age comparison (Mann–Whitney *U* test); Gender comparison (chi‐squared test).

Using the Mann–Whitney *U* test, we compared PEOU and PU scores before and after crossover. The results indicated that the PEOU scores of Group B were significantly higher than those of Group A after crossover (Z = −2.34, *p* = 0.02). Although the PEOU scores of Group B were higher than those of Group A before crossover, the difference was not statistically significant. (Z = −1.53, *p* = 0.13). Confidence and self‐satisfaction with DO training in Group B generally higher than in Group A, with Group B showing significantly higher confidence in session one (Z = −2.05, *p* = 0.04). (Table 2).

**TABLE 2:** Comparison of PEOU, PU, confidence of direct ophthalmoscopy performance and self‐satisfaction of the training between Groups (A and B) before and after crossover design.

		Group A (*n* = 40) Median (IQR)	Group B (*n* = 39) Median (IQR)	Z	*p* *
Session one	PEOU	17 (14, 20)	19 (17, 20)	−1.529	0.13
	PU	20 (17.5, 23)	21 (18, 24)	−0.705	0.48
	Confidence	19.5 (16.5, 24)	23 (20, 25)	−2.045	**0.04**
	Self‐satisfaction	11 (9, 13)	11 (11, 12)	−0.586	0.55
Session two	PEOU	18.5 (16, 21)	20 (19, 22)	−2.343	**0.02**
	PU	20 (18, 23)	20 (20, 23)	−1.116	0.26
	Confidence	23 (20, 25)	24 (22, 26)	−1.382	0.16
	Self‐satisfaction	12 (11, 13)	12 (12, 14)	−0.943	0.35

Abbreviations: PEOU, perceived ease of use; PU, perceived usefulness.

* Results conducted by Mann–Whitney *U* test.

Intragroup comparisons showed significant improvement in PEOU, confidence and self‐satisfaction after session two (*p* < 0.05, Table 3). However, PU results were inconsistent (*p* > 0.05). Satisfaction of students with PFC assistance was notably high. More than 75% of students rated 4 or above points on all six questions (Figure 3).

**TABLE 3:** Intragroups comparison of PEOU and PU between two sessions.

		Session one median (IQR)	Session two median (IQR)	Z	*p* *
Group A	PEOU	17 (14, 20)	18.5 (16, 21)	−3.446	**< 0.001**
	PU	20 (17.5, 23)	20 (18, 23)	−0.621	0.534
	Confidence	19.5 (16.5, 24)	23 (20, 25)	−3.432	**< 0.001**
	Self‐satisfaction	11 (9, 13)	12 (11, 13)	−2.727	**< 0.001**
Group B	PEOU	19 (17, 20)	20 (19, 22)	−3.367	**< 0.001**
	PU	21 (18, 24)	20 (20, 23)	0.176	0.86
	Confidence	23 (20, 25)	24 (22, 26)	−2.569	**0.01**
	Self‐satisfaction	11 (11, 12)	12 (12, 14)	−2.898	**< 0.001**

Abbreviations: PEOU, perceived ease of use; PU, perceived usefulness.

* Results conducted by Wilcoxon signed‐rank test.

**FIGURE 2:**
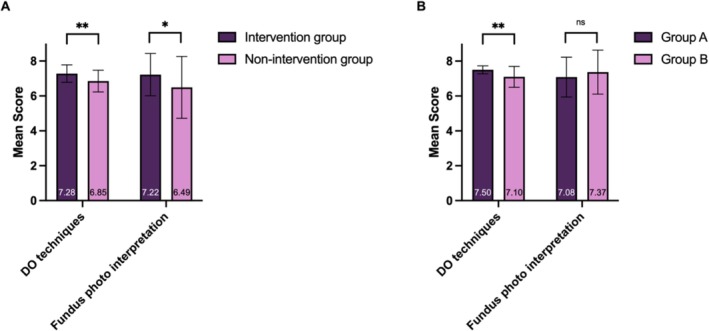
**Comparisons of assessment results between Groups A and B and intervention and non‐intervention groups.** Starred data marks statistically significant data (*p < 0.05, **p < 0.001); Error bars show standard deviation.

The intervention group was compared with the previous non‐intervention group (Figure 2A). For the intervention group, the mean scores of DO technical and fundus photo assessments were 7.28 ± 0.50 and 7.22 ± 1.21, respectively. For non‐intervention group, the mean scores were 6.85 ± 0.62 and 6.49 ± 1.77, respectively. Independent *t*‐tests showed significantly higher scores in the intervention group (TDO technical assessment: *t* = 5.11, *p* < 0.001; fundus photo assessment: *t* = 3.17, *p* = 0.002). Between Groups A and B, TDO technical assessment of Group A was significantly better than Group B (*t* = 4.1951, *p* < 0.001), but not showing significant difference for fundus photointerpretation assessment (Figure 2B).

**FIGURE 3:**
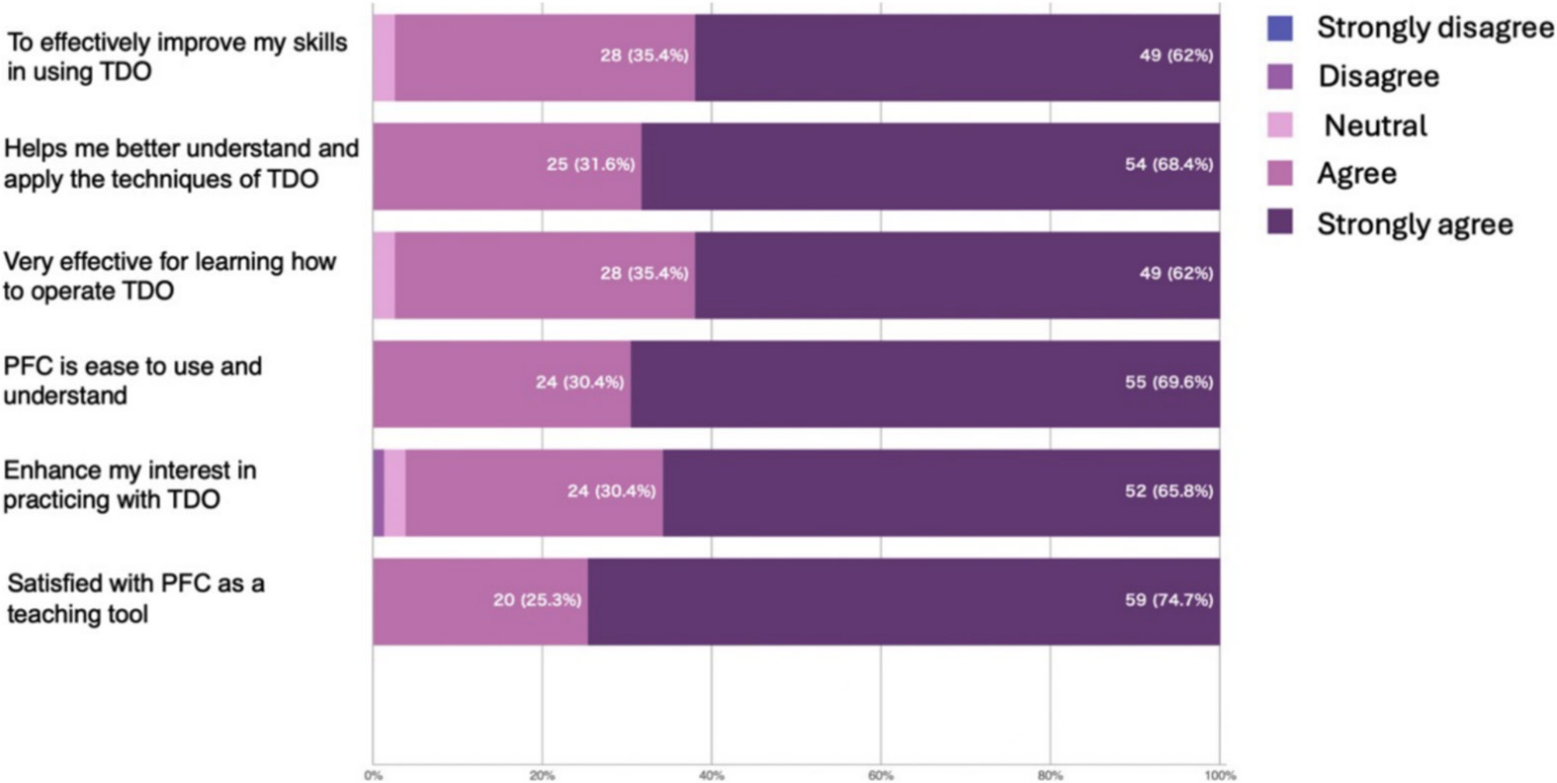
Satisfaction of PFC‐assisted teaching method.

*Satisfaction of students with PFC assistance was notably high*.

### 3.1 Ethics Statement

The study adhered to the Declaration of Helsinki and approval was granted by the Ethical Review Institute of Zhongshan Ophthalmic Center.(2024KYPJ064) The research protocol was approved by the Institutional Review Board/Ethics Committee of Zhongshan Ophthalmic Center.

## 4 Implications

Although TDO is an integral part of physical examinations, the rise of user‐friendly digital devices has reduced reliance on it due to technical and interpretative challenges [12]. In this study, PFC served as an adjunctive teaching tool for TDO. We compared the effectiveness of PFC‐assisted teaching with TDO teaching during clerkship. PFC assistance could increase overall performance in objective assessments, including DO performance and fundus photo interpretation. Students taught with PFC gained more confidence in learning DO techniques and PEOU, especially with earlier exposure. Most students were satisfied with PFC assistance for learning DO techniques, supporting our hypothesis mentioned.

Our study found better results in the final DO techniques and fundus photo assessments using PFC‐assisted intervention. One possible reason is that PFC enhances the learning experience during DO practice, boosting student enthusiasm. PFC, like fool‐style fundoscopy, follows the similar processing principles of TDO and allows for a wider field of view. Students can capture fundus photos themselves and review them, reducing the learning curve. The photos also help identify and recognise magnified fundal signs, minimising uncertainty and building confidence [7]. Besides, PFC reduces the discomfort and demotivation caused by prolonged novice examination. Interestingly, Group A, which experienced PFC in session two, had significantly higher scores on DO technique assessment than Group B. This finding indicated that there was uncertainty in visualising the fundus, which caused low confidence in session one. Later experiences with PFC assistance could further confirm their fundamental DO techniques.

*Our study found better results in the final DO techniques and fundus photo assessments using PFC‐assisted intervention*.

In this study, cumulative learning effect of training significantly increased PEOU, confidence and self‐satisfaction. The PFC assistance was attractive and psychologically beneficial for enhancing students' motivation and interest, in addition to objective scores. However, PU was not significantly different in the time domain and between Groups A and B in our study. One possible reason is the short duration of the ophthalmology clerkship. DO was new to fourth‐year students with limited clinical exposure influenced by a culture of incompetency and clinical practice patterns [7]. PEOU and PU consistently account for intended usage and actual behaviours of premedics [13]. Our findings indicated that DO taught using the PFC assistance could notably increase the PEOU of DO.

*The PFC assistance was attractive and psychologically beneficial for enhancing students' motivation and interest, in addition to objective scores*.

Students were predominantly favourable towards PFC as an adjunctive tool for DO learning.(Figure 2) In particular, all students agreed or strongly agreed with statements about PFC as helpful in understanding and applying DO, since PFC is easy to use and understand, and they were also satisfied with PFC as a teaching tool for DO.

Digital evolution modified lots of the clinical and medical education settings. The art of DO should not be forgotten as it is still an assessable and affordable tool in some critical clinical environments. It trains the fundamental examination ability of medics. The study was conducted in a crossover setting, which provided fairness to student groups and minimised biases such as recall bias. Besides, our study was first to investigate the changes in PEOU and PU of DO via the PFC assistance with modified Technology Acceptance Model [13]. We also used a rigorous questionnaire to predict the future use of DO under the new teaching method with excellent internal reliability.

*Our findings indicated that DO taught using the PFC assistance could notably increase the PEOU of DO*.

### 4.1 Limitations

Firstly, it was a short‐term study conducted in a weekly clerkship. Further longitudinal investigations are needed to prove the long‐term effects of interventions. Furthermore, the sample size should be larger to enhance representativeness.

## 5 Conclusions

The PFC‐assisted teaching method is highly satisfied by the premedics and is beneficial for learning DO. PEOU and confidence of students on DO might be favourable to reinvigorate wanned use of DO in the future.

## Author Contributions

**Ching‐Kit Tsui:** writing – original draft, conceptualization, methodology, formal analysis, software, data curation, visualization, writing – review and editing, validation. **Yuxian Zou:** methodology, supervision, project administration, resources, investigation. **Zhenzhen Liu:** project administration, supervision. **Rongxin Chen:** funding acquisition, supervision, project administration, writing – review and editing, formal analysis. **Yiqing Li:** project administration, supervision.

## Conflicts of Interest

The authors declare no conflicts of interest.

## Data Availability

The data that support the findings of this study are available from the corresponding author upon reasonable request.

## References

[1] M. J. Burton , J. Ramke , A. P. Marques , et al., “The Lancet Global Health Commission on Global Eye Health: Vision Beyond 2020,” Lancet Global Health 9, no. 4 (2021): e489–e551. 33607016 10.1016/S2214-109X(20)30488-5 PMC7966694

[2] T. Y. Chan , A. S. Rai , E. Lee , J. T. Glicksman , and C. M. Hutnik , “Needs Assessment of Ophthalmology Education for Primary Care Physicians in Training: Comparison With the International Council of Ophthalmology Recommendations,” Clinical Ophthalmology 5 (2011): 311–319. 21468339 10.2147/OPTH.S17567 PMC3065573

[3] E. Roberts , R. Morgan , D. King , and L. Clerkin , “Funduscopy: A Forgotten art?,” Postgraduate Medical Journal 75, no. 883 (1999): 282–284. 10533632 10.1136/pgmj.75.883.282 PMC1741240

[4] I. H. Yusuf , J. F. Salmon , and C. K. Patel , “Direct Ophthalmoscopy Should Be Taught to Undergraduate Medical Students—Yes,” Eye 29, no. 8 (2015 Aug): 987–989. 26043702 10.1038/eye.2015.90 PMC4541359

[5] International Task Force on Opthalmic Education of Medical Students, International Council of Opthalmology , “Principles and Guidelines of a Curriculum for Ophthalmic Education of Medical Students,” Klinische Monatsblätter für Augenheilkunde 223, no. Suppl 5 (2006): S1–S19. 10.1055/s-2006-951844 17238059

[6] B. R. Straatsma , G. J. Coscas , G. O. H. Naumann , B. E. Spivey , H. R. Taylor , and M. O. M. Tso , “International Ophthalmology Strategic Plan to Preserve and Restore Vision—Vision for the Future,” American Journal of Ophthalmology 132, no. 3 (2001): 403–404. 11530055 10.1016/s0002-9394(01)01137-0

[7] H. P. Dunn , C. J. Kang , S. Marks , S. M. Dunn , P. R. Healey , and A. J. White , “Optimising Fundoscopy Practices Across the Medical Spectrum: A Focus Group Study,” PLoS ONE 18, no. 1 (2023): e0280937. 36706098 10.1371/journal.pone.0280937 PMC9882965

[8] H. Wang , X. Liao , M. Zhang , C. P. Pang , and H. Chen , “Smartphone Ophthalmoscope as a Tool in Teaching Direct Ophthalmoscopy: A Crossover Randomized Controlled Trial,” Medical Education Online 28, no. 1 (2023): 2176201. 36762913 10.1080/10872981.2023.2176201 PMC9930769

[9] M. Chen , C. Swinney , M. C. Bs , M. B. Bs , and A. N. Bs , “Comparing the Utility of the Non‐Mydriatic Fundus Camera to the Direct Ophthalmoscope for Medical Education,” Public Health 74, no. 3 (2015): 93. PMC4363930 25821651

[10] J. Kohler , T. M. Tran , S. Sun , and S. R. Montezuma , “Teaching Smartphone Funduscopy With 20 Diopter Lens in Undergraduate Medical Education,” Clinical Ophthalmology 15 (2021): 2013–2023. 34012252 10.2147/OPTH.S266123 PMC8128496

[11] F. D. Davis , “Perceived Usefulness, Perceived Ease of Use, and User Acceptance of Information Technology,” MIS Quarterly 13, no. 3 (1989): 319–344.

[12] D. D. Mackay , P. S. Garza , B. B. Bruce , N. J. Newman , and V. Biousse , “The Demise of Direct Ophthalmoscopy,” Neurology: Clinical Practice 5, no. 2 (2015): 150–157. 26137422 10.1212/CPJ.0000000000000115 PMC4404284

[13] V. Venkatesh and F. D. Davis , “A Theoretical Extension of the Technology Acceptance Model: Four Longitudinal Field Studies,” Management Science 46, no. 2 (2000): 186–204.

